# Detection and characterisation of Xpert CT/NG assay *Neisseria gonorrhoeae* diagnostic escape mutants, England, June 2025

**DOI:** 10.2807/1560-7917.ES.2025.30.36.2500673

**Published:** 2025-09-11

**Authors:** Melissa Jansen van Rensburg, Rachel Pitt-Kendall, Michelle Hincke, Penelope R Cliff, Jonathan Shaw, Katy Sinka, John Saunders, Helen Fifer, Sarah Alexander

**Affiliations:** 1 Sexually Transmitted Infections Reference Laboratory, UK Health Security Agency, London, United Kingdom; 2 Royal Devon University Healthcare NHS Foundation Trust, Exeter, United Kingdom; 3 Blood Safety, Hepatitis, STI & HIV Division, UK Health Security Agency, London, United Kingdom

**Keywords:** Cepheid, Xpert CT/NG Assay, diagnostic escape mutant

## Abstract

We report the timely detection of a strain of *Neisseria gonorrhoeae* which was confirmed as a diagnostic escape mutant on the Cepheid Xpert CT/NG assay in England in June 2025. The reason for assay failure was a likely recombination event with *Neisseria meningitidis*, which removed both assay target sites. Seven historical putative Xpert CT/NG assay diagnostic escape mutants were also identified following subsequent in silico screening of gonococcal genome collections, but currently there is no evidence of widespread circulation.

In June 2025, an isolate of *Neisseria gonorrhoeae* that escaped detection on the Cepheid GeneXpert CT/NG assay was referred to the UK Health Security Agency Sexually Transmitted Infections Reference Laboratory from a primary diagnostic laboratory in England. Genomic analysis confirmed this isolate was lacking the genomic target sites for this assay, due to a likely recombination event with *Neisseria meningitidis*. 

## Detection of *Neisseria gonorrhoeae* with the Xpert CT/NG assay

The Cepheid GeneXpert (Xpert) CT/NG assay is a rapid diagnostic test for dual detection of *Chlamydia trachomatis* (CT) and *Neisseria gonorrhoeae* (NG) in clinical specimens [1]. The NG component of the assay targets two NG chromosomal sequences, designated NG2 and NG4, to facilitate detection and confirmation in one test. The Xpert assay requires both NG2 and NG4 targets to report amplification to return a positive result. *Neisseria gonorrhoeae* is known to recombine with other *Neisseria* species [2], complicating diagnostic algorithms [3]. The Xpert targets are thought to be conserved and specific to NG, and they are entirely (NG2) or partially (NG4) absent from other *Neisseria*, including *Neisseria meningitidis* (NM) [4,5].

## Case detection and laboratory results

In June 2025, the national Sexually Transmitted Infections Reference Laboratory (STIRL), UK Health Security Agency (UKHSA), confirmed the identification of an NG strain that was negative for both NG2 and NG4 Xpert targets. The strain was isolated from an asymptomatic female who reported sex with two European male partners during recent travel in the Asia-Pacific region. The individual attended a sexual health service as an NG contact, following partner notification. A detailed clinical review identified no risk of onward transmission. 

Nucleic acid amplification test (NAAT) specimens from pharyngeal and vaginal sites were positive on the Roche Cobas 6800 CT/NG (Ct values: 23.56 and 25.16, respectively) but negative on the Xpert (both targets), which was used as a confirmatory test. Both pharyngeal and endocervical sites had positive NG cultures and both isolates were negative on the Xpert but positive on the Roche Cobas assays. The endocervical isolate (H25–379) was referred to STIRL, where additional examination found it to be positive on the GeneProof and Hologic assays (Aptima Combo 2 and the NG Assay), and the MALDI-TOF gave a high-confidence species level identification. Susceptibility testing via ETEST (BioMerieux, France) indicated susceptibility to ceftriaxone, cefixime, azithromycin, ciprofloxacin, benzylpenicillin and tetracycline.

## Genomic analyses

The isolate H25–379 was sequenced, as previously described [6]. An in-house bioinformatics pipeline was run to: confirm species identification [7]; generate a de novo assembly [8]; and determine sequence types (STs) for multilocus sequence typing (MLST), NG sequence typing for antimicrobial resistance (NG-STAR), and NG multi-antigen sequence typing (NG-MAST). BLAST [9] was used to query H25–379 for NG2 and NG4 using nucleotide sequences from the Xpert patent [10]. Neither target was detected.

Two gonococcal genome collections were screened for additional isolates lacking NG2 and/or NG4: (i) 6,118 genomes from England and Wales received by UKHSA as part of the Gonococcal Resistance to Antimicrobial Surveillance Programme (GRASP; 2020 and 2022–24) [11], and (ii) a global dataset of 24,551 genomes accessed via the *Neisseria* PubMLST database [12] (inclusion criteria: < 400 contigs, N_50_ > 30,000 bp, total size 1.95–2.5 Mbp). In total, 2 of 6,118 (0.03%) GRASP isolates lacked either NG2 (20GRASP1107) or NG4 (20GRASP0157) (Table). Four (0.02%) genomes from the global dataset lacked both targets (NGPT20091; NGPT20099; NGPT23165; ERR3578062), one of which was also from England and collected as part of GRASP in 2016 (ERR3578062, Table). One PubMLST isolate lacked NG4 (FR20–026)(Table), and five (0.02%) had partial NG2 or NG4 sequences, all of which were close to a contig break. Isolates with partial NG2 or NG4 sequences were not investigated further (PubMLST identifiers: 53556, 56151, 108476, 109237 and 27354).

**Table:** Details of gonococcal genomes identified in GRASP^a^ and PubMLST^b^ datasets as lacking Xpert targets NG2 and/or NG4, 2016–2025 (n = 8)

Data source	Isolate	PubMLST identifier	Country	Year	Patient information			BLAST result		Sequence type		
					Sex	Sexual orientation	Specimen site	NG2	NG4	MLST	NG-STAR	NG-MAST
Referred isolate	H25–379	170406	England	2025	Female	Heterosexual	Endocervical	ND	ND	1596	4411	Novel
GRASP^a^	20GRASP0157	170833	England	2020	Male	Heterosexual	Rectal	Full length	ND	10314	1615	21872
	20GRASP1107	170832	England	2020	Male	Unknown	Rectal	ND	Full length	15679	165	6388
*Neisseria* species PubMLST database^b^	ERR3578062 [15]	107354	England	2016	Male	GBMSM	Rectal	ND	ND	1596	4411	5016
	FR20–026 [16]	156586	France	2020	Unknown	Unknown	Unknown	Full length	ND	10314	1615	21872
	NGPT20091	164007	Portugal	2020	Male	Unknown	Unknown	ND	ND	1588	3403	964
	NGPT20099	164013	Portugal	2020	Female	Unknown	Unknown	ND	ND	1588	1873	338
	NGPT23165	164445	Portugal	2023	Male	Unknown	Unknown	ND	ND	8123	4621	20610

GBMSM: gay, bisexual and other men who have sex with men; GRASP: Gonococcal Resistance to Antimicrobial Surveillance Programme; MLST: multilocus sequence typing; ND: not detected; NG: *Neisseria gonorrhoeae*; NG-MAST: NG multi-antigen sequence typing; NG-STAR: NG sequence typing for antimicrobial resistance.

^a^ A dataset of 6,118 genomes from England and Wales received by UKHSA as part of the GRASP collection (2020 and 2022–24) [11].

^b^ A global dataset of 24,551 genomes accessed via the *Neisseria* PubMLST database [12] with inclusion criteria: < 400 contigs, N_50_ > 30,000 bp, total size 1.95–2.5 Mbp.

**Figure 1:**
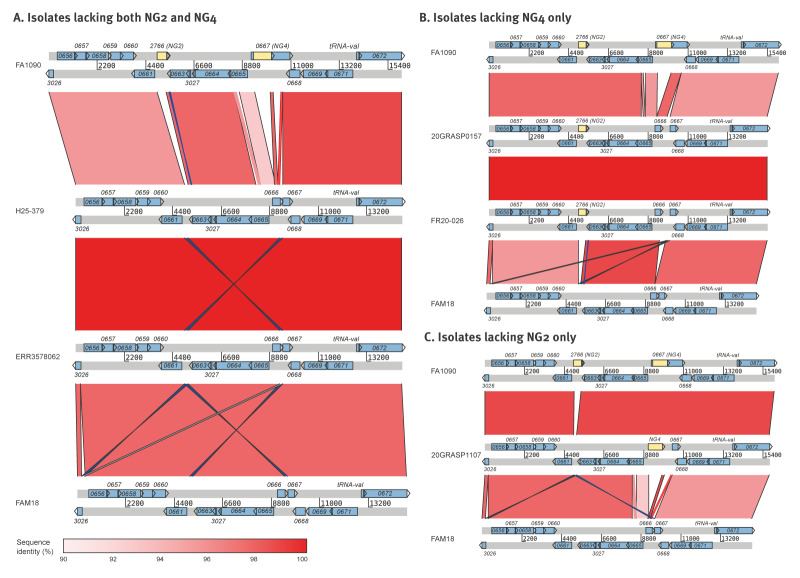
Genomic comparison of the Xpert target loci and flanking sequences in genomes lacking NG2 and/or NG4 from GRASP and PubMLST datasets and the referred isolate, 2016–2025 (n = 5)

NG: *Neisseria gonorrhoeae*; NM: *Neisseria meningitidis*. 

**Figure 2:**
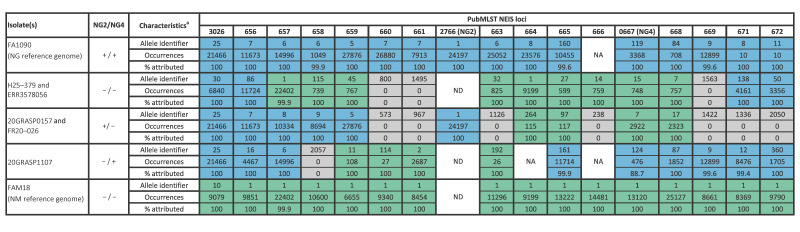
Species assignment of PubMLST NEIS3026–NEIS0672 alleles in reference genomes for *Neisseria gonorrhoeae* (FA1090) and *Neisseria meningitidis* (FAM18), and five gonococcal genomes lacking Xpert targets NG2 and/or NG4, 2016–2025 (n = 5)

NG2 and NG4 were ca 4 kb apart in the NG reference genome FA1090 (GenBank accession: AE004969). Therefore, the region spanning PubMLST loci NEIS3026 and NEIS0672 (encompassing both targets and ca 5 kb flanking sequences) was examined in genomes lacking NG2 and/or NG4. This region was not detected in NGPT20091, NGPT20099, and NGPT23165, but sequences from the remaining five isolates were queried against NCBI. The top hit was consistently NM, so these sequences were compared with reference genomes NG FA1090 and NM FAM18 (GenBank accession: AM421808) (Figure 1). Gene content and order were conserved between FA1090 and FAM18, except for the target sequences, and sequence identity across the region ranged from 92 to 98%. Patterns of species-specific alleles across NEIS3026–NEIS0672 in isolates lacking NG2 and/or NG4 provided further support for recombination with NM (Figure 2). Distinct putative recombination fragments were identified in the three sets of mutants (Figure 1 and 2). The 14.8 kb sequences from the double mutant strains H25–379 and ERR3578062 were identical (Figure 1A). Despite being collected 9 years apart, these strains were indistinguishable by MLST and NG-STAR, although their NG-MAST profiles differed at *tbpB* (Table). The 15.4 kb sequences from NG4-negative isolates 20GRASP0157 and FR20–026 were also identical (Figure 1B). These isolates were collected in 2020 in England and France and corresponded to the same STs across all three typing schemes (Table).

Isolates lacking both NG2 and NG4 (panel 1A), NG4 only (panel 1B), or NG2 only (panel 1C) are compared with reference genomes NG FA1090 and NM FAM18. Forward and reverse strands are illustrated as grey bars, and blue boxes represent PubMLST loci. Italicised numeric annotations correspond to PubMLST locus identifiers NEIS3026-NEIS0672. The target sequences NG2 and NG4 are shown in yellow, partially overlapping with NEIS2766 and NEIS0667, respectively. Red segments connecting isolates indicate pairwise nucleotide sequence similarity with colour intensity corresponding to percentage identity, as shown in the legend. Divergent sequences are unconnected and unshaded. Inverted sequences are overlaid in dark blue. Figures were generated with the Artemis Comparison Tool [17] with downstream editing in Inkscape.

minus (−): absent; NA: no allele assigned; ND: not detected; NG: *Neisseria gonorrhoeae*; NM: *Neisseria meningitidis*; plus (+) : present.

^a^ For each isolate, the allele identifier, the number of occurrences in the PubMLST dataset and the percentage (%) attributed to predominant species for different NEIS loci are provided.

PubMLST NEIS loci including NG2, NG4 and ca 5 kb flanking regions were analysed. Shading indicates the predominant species associated with a given allele: blue, NG; green, NM; grey, allele restricted to isolate(s) of interest. Allele counts and proportions were determined based on genomes of 28453 NG, 45582 NM, and 1777 *Neisseria* species isolates available in the *Neisseria* species PubMLST isolate database.

## Discussion

A strain of NG which produced false negative results on the Xpert CT/NG assay was isolated in England in June 2025. Genomic analyses determined that assay failure was caused by the loss of both target sequences resulting from likely recombination with *N. meningitidis*. Subsequent in silico analysis of two large gonococcal genome datasets revealed a further seven historical isolates which theoretically would also give rise to false negative results on the Xpert assay. Despite some genomes being publicly available since 2019, these putative escape mutants have not previously been highlighted. The most notable finding was a second English mutant strain from 2016, which had an identical recombination fragment and was indistinguishable from the referred isolate based on MLST and NG-STAR, providing evidence that this strain had been circulating for at least 9 years. Both isolates corresponded to MLST ST1596, a relatively rare ST among gonococcal genomes in the GRASP collection. Phenotypic antimicrobial susceptibility data demonstrated both isolates were fully susceptible to the antimicrobials tested.

Two further potential escape mutant strains (20GRASP0157 and 20GRASP1107) were identified in the GRASP genomic dataset, which lacked NG4 and NG2, respectively. Concerningly, the isolates are genomically different from both each other and the original referred isolate, H25–379. This provides evidence that this region of the gonococcal genome has undergone recombination with *N. meningitidis* on at least three independent occasions.

The referred diagnostic escape mutant H25–379 was fortuitously detected, which can in part be attributed to (i) UK NG management guidelines recommending that culture be attempted from all patients with positive molecular NG results (primarily for antimicrobial resistance testing) [13]; (ii) the robust local confirmatory testing algorithm in place within the primary diagnostic laboratory; and (iii) the English public health system having an active reference laboratory that investigates anomalous and discordant NG culture results. These factors in combination with strong gonococcal surveillance systems and large historical genomic datasets ensure that when such diagnostic mutant(s) are identified the potential impact can be rapidly assessed.

The precise prevalence of these Cepheid Xpert escape mutant(s) within England is currently unknown. Since 2022, whole genome sequencing has been routinely performed for all isolates collected as part of GRASP (ca 1,500 annually). This sequencing accounts for ca 2% of the annual NG diagnoses in England (2024 diagnoses: 71,802 [14]). Therefore, it can be predicted that at the present time it is unlikely that these diagnostic escape mutant(s) are present in the English gonococcal population at a high enough level to compromise the routine performance of the Cepheid Xpert test. A prospective case finding exercise will be required to determine the true prevalence of these mutant(s). 

## Conclusion

We have detected an NG Cepheid Xpert escape mutant in England. This strain lacks both the target sites for the affected assay, due to a likely recombination event which has occurred with *N. meningitidis*. Whilst we predict that these mutant(s) are rare, UK and global clinics and laboratories should remain vigilant for any unusual diagnostic scenarios when using the Cepheid Xpert CT/NG assay.

## Data Availability

Genome assemblies for isolates newly described in this report are available in the *Neisseria* species PubMLST isolate database under identifiers 170406 (H25-379), 170833 (20GRASP0157), and 170832 (20GRASP1107) (https://pubmlst.org/bigsdb?db=pubmlst_neisseria_isolates).
