# Addendum for Euro Surveill. 2025;30(36)

**DOI:** 10.2807/1560-7917.ES.2025.30.40.251009a

**Published:** 2025-10-09

**Authors:** Eurosurveillance editorial team

**Affiliations:** 1 European Centre for Disease Prevention and Control (ECDC), Stockholm, Sweden

In the article ‘Detection and characterisation of Xpert CT/NG assay *Neisseria gonorrhoeae* diagnostic escape mutants, England, June 2025’ by M Jansen Van Rensburg et al., published on 11 September 2025, one confirmed and seven putative Cepheid Xpert CT/NG escape mutants were described (three English, three Portuguese, and one French). Following communication with the Portuguese NIH, read mapping confirmed that the lack of Xpert targets NG2 and NG4 in the three Portuguese genomes was an assembly artefact in the data submitted to the PubMLST database and these targets were subsequently detected using the sequencing reads. Further read mapping, endorsed the results obtained for the remaining four genomes (20GRASP0157; 20GRASP1107; ERR3578062; FR20–026) and the referred strain (H25–379), all of which were confirmed to be lacking NG2 and/or NG4 and would be expected to return a false negative result on the Xpert CT/NG assay.

